# AI improves accuracy, agreement and efficiency of pathologists for Ki67 assessments in breast cancer

**DOI:** 10.1038/s41598-024-51723-2

**Published:** 2024-01-13

**Authors:** Amanda Dy, Ngoc-Nhu Jennifer Nguyen, Julien Meyer, Melanie Dawe, Wei Shi, Dimitri Androutsos, Anthony Fyles, Fei-Fei Liu, Susan Done, April Khademi

**Affiliations:** 1 Electrical, Computer, and Biomedical Engineering, Toronto Metropolitan University, Toronto, ON Canada; 2 Department of Laboratory Medicine and Pathobiology, University of Toronto, Toronto, ON Canada; 3 School of Health Services Management, Toronto Metropolitan University, Toronto, ON Canada; 4 Princess Margaret Cancer Centre, University Health Network, Toronto, ON Canada; 5 Keenan Research Center for Biomedical Science, St. Michael’s Hospital, Unity Health Network, Toronto, ON Canada; 6 Institute for Biomedical Engineering, Science Tech (iBEST), A Partnership Between St. Michael’s Hospital and Toronto Metropolitan University, Toronto, ON Canada; 7 Vector Institute for Artificial Intelligence, Toronto, ON Canada; 8 Department of Medical Imaging, University of Toronto, Toronto, ON Canada

**Keywords:** Breast cancer, Cancer imaging, Tumour biomarkers, Cancer, Biomarkers, Biomarkers, Translational research

## Abstract

The Ki-67 proliferation index (PI) guides treatment decisions in breast cancer but suffers from poor inter-rater reproducibility. Although AI tools have been designed for Ki-67 assessment, their impact on pathologists' work remains understudied. 90 international pathologists were recruited to assess the Ki-67 PI of ten breast cancer tissue microarrays with and without AI. Accuracy, agreement, and turnaround time with and without AI were compared. Pathologists’ perspectives on AI were collected. Using AI led to a significant decrease in PI error (2.1% with AI vs. 5.9% without AI, *p* < 0.001), better inter-rater agreement (ICC: 0.70 vs. 0.92; Krippendorff’s α: 0.63 vs. 0.89; Fleiss’ Kappa: 0.40 vs. 0.86), and an 11.9% overall median reduction in turnaround time. Most pathologists (84%) found the AI reliable. For Ki-67 assessments, 76% of respondents believed AI enhances accuracy, 82% said it improves consistency, and 83% trust it will improve efficiency. This study highlights AI's potential to standardize Ki-67 scoring, especially between 5 and 30% PI—a range with low PI agreement. This could pave the way for a universally accepted PI score to guide treatment decisions, emphasizing the promising role of AI integration into pathologist workflows.

## Introduction

Ki-67 immunohistochemistry (IHC) serves as a reliable marker of cell proliferation and is widely used to evaluate the aggressiveness and prognosis of human tumors. Notably, Ki-67 has been adopted for prognostication in breast cancer, with elevated Ki-67 expression correlating with poorer prognosis^1,2^. The Ki-67 proliferation index (PI) in breast cancer is a measure of the percentage of tumor cells with nuclear immunoreactivity relative to the total number of malignant cells assessed^3^. A meta-analysis of 64,196 patients revealed that higher Ki-67 PI values are associated with worse overall survival in breast cancer, with 25% being a cutoff of strong outcome prognostication^4^.

The monarchE committee reported that among patients with early-stage HR+, HER2− breast cancer, and nodal involvement, the addition of abemaciclib to hormone therapy significantly improves cancer-specific free survival and decreases the risk of disease recurrence^5–7^. For tumor stage 1 to 2, nodal stage 0 to 1, ER+/HER2− breast cancer, the International Ki-67 in Breast Cancer Working Group’s (IKWG) consensus in 2021 recommended using Ki-67 to aid in the decision-making of adjuvant chemotherapy only for cases with a very low (< 5%) or very high (> 30%) PI due to substantial inter-rater variability within this range^8,9^. The panelists of the St. Gallen International Consensus Conference in 2021 generally support this recommendation^10^. The monarchE phase III clinical trial studied the impact of a high Ki-67 PI on disease recurrence in a cohort of patients with HR+/HER2− node-positive breast cancer with high-risk clinicopathological features (at least 4 positive lymph nodes, or 1 to 3 positive lymph nodes with either tumor size ≥ 5 cm or histological grade 3 disease). Their analyses demonstrated that a Ki-67 PI ≥ 20% in patients treated with endocrine therapy alone was associated with a significantly increased risk of recurrence within three years compared to patients with lower Ki-67 expression^6,11^. Following this, the American Food and Drug Administration and Health Canada approved the use of abemaciclib (CDK4/6 inhibitor) for patients with HR+/HER2− high-risk early breast cancer and a Ki-67 PI of ≥ 20%^12^. In a recently published landmark study^13^ based on 500 patients, it was demonstrated that a PI score threshold of < 13.25% derived from Ki-67 slides effectively identified women with luminal A breast cancer who could be safely treated without local breast radiation therapy. This underscores the clinical significance of Ki-67 as a marker with significant promise in guiding management decisions for breast cancer patients.

The current gold standard for quantifying Ki-67 PI is to manually evaluate at least 500 malignant cells based on IKWG recommendations^8,9^. However, this method is labor-intensive, time-consuming, and prone to poor inter-rater reproducibility and errors^14,15^. As a result, it is hard to standardize and use Ki-67 for clinical assessments. As shown in the recommendations from the IKWG^8,9^, the assessment by the pathologist is most reliable for PI values below 5% and above 30% (the 5 to 30% range is subject to the most interpretation variability). The Canadian Association of Pathologists recommends that a second pathologist assess PIs in this range, or use a computer assessment tool to improve robustness^12^. Considering this range is critical for treatment decisions, its reliability must be improved. The recent emergence of digital pathology and high-performance AI algorithms offers the possibility that automated PI scoring can overcome these challenges by accurately and efficiently measuring cell count. There have been several AI-based Ki-67 assessment tools developed^16–21^, and the advantages are becoming increasingly evident.

Several comparative studies have reported the role of AI-assisted assessments of Ki-67 PI in breast cancer^19,22,32^. These studies demonstrated that AI-aided assessment of Ki-67 could achieve a lower mean error^23^ and a lower standard error deviation^19^, however, the impact on inter-rater agreement is less clear. Additionally, while these studies have encompassed broad PI ranges from 0 to 100%, the effect of AI assistance in the clinically crucial 5 to 30% PI interval has not yet been studied.

Herein, we conducted a large-scale, international study that analyzed the effects of AI assistance on key aspects of pathologists' work, including accuracy, inter-rater agreement, and turnaround time (TAT) in the context of Ki-67 scoring for breast cancer. Our focus was on assessing these metrics within the 5 to 30% PI range to better understand the implications and usability of AI-assisted Ki-67 evaluations. Additionally, we gathered insights into pathologists' perspectives, trust levels, and readiness to adopt AI technologies, highlighting the importance of user acceptance. This study provides a strong foundation for understanding the future impact and potential of AI tools for Ki-67 scoring in the daily routine of pathologists.

## Materials and methods

Ethics approval for the study was obtained from Toronto Metropolitan University (REB: 2022-154). All experiments were performed in accordance with the Tri-Council policy statement 2 for the ethical conduct of research involving humans.

### Case selection, TMA preparation, and image acquisition

A subset of ten TMAs from the Toronto-British Columbia trial was used for this study^24^, which was composed of node-negative patients above the age of 50 years with invasive breast cancer^25^. Tissue microarrays (TMAs) were constructed using a 0.6 mm tumor core procured from formalin-fixed, paraffin-embedded specimens. TMA sections, with a thickness of 0.5 μm, were stained using a 1:500 dilution of SP6 (ThermoFisher Scientific, Waltham, MA, USA)—a Ki-67 antibody—and counterstained with hematoxylin. The study incorporated ten TMAs with high tumor cellularity, averaging 2093 neoplastic cells per TMA and a PI range of 7 to 28%^16^. This range, which poses a challenge for pathologists, encompasses the clinically relevant PI cutoffs identified in prior studies^6,11,13^.

### AI tool

A deep learning-based AI tool for IHC quantification, UV-Net, developed by Toronto Metropolitan University, was used in the study^18^. This tool detects neoplastic cells in IHC-stained tissue and differentiates Ki-67 positive from Ki-67 negative tumor cells. Its underlying architecture, a modified U-Net, includes additional connections for densely packed nuclei and replaces the standard 3 × 3 convolutional layers with 'V-Blocks'. These V-Block connections maintain high-resolution nuclear features for precise differentiation between nuclei; each V-Block inputs n channels and outputs 2n channels, forming a 'V' shape across four successive stages.

The AI tool was trained using 256 × 256 RGB patches of WSIs from St. Michael's Hospital, and an open-source dataset "Deepslides"^26^ from × 20 Aperio AT Turbo and × 40 Aperio ScanScope scanners respectively. Images were annotated with single-pixel centroid markers distinguishing Ki-67 positive and Ki-67 negative tumor nuclei cells^20^, defining positive nuclei as any brown color above the background, following the IKWG’s recommendations^8,9^. Single-pixel markers were extended into circular areas using a Gaussian function, this allocated the highest value to the center of the nuclei, incorporated more contextual information, and improved the efficiency of the training process. A Huber loss function was used to regress and predict the centroid of nuclei.

**Figure 1:**
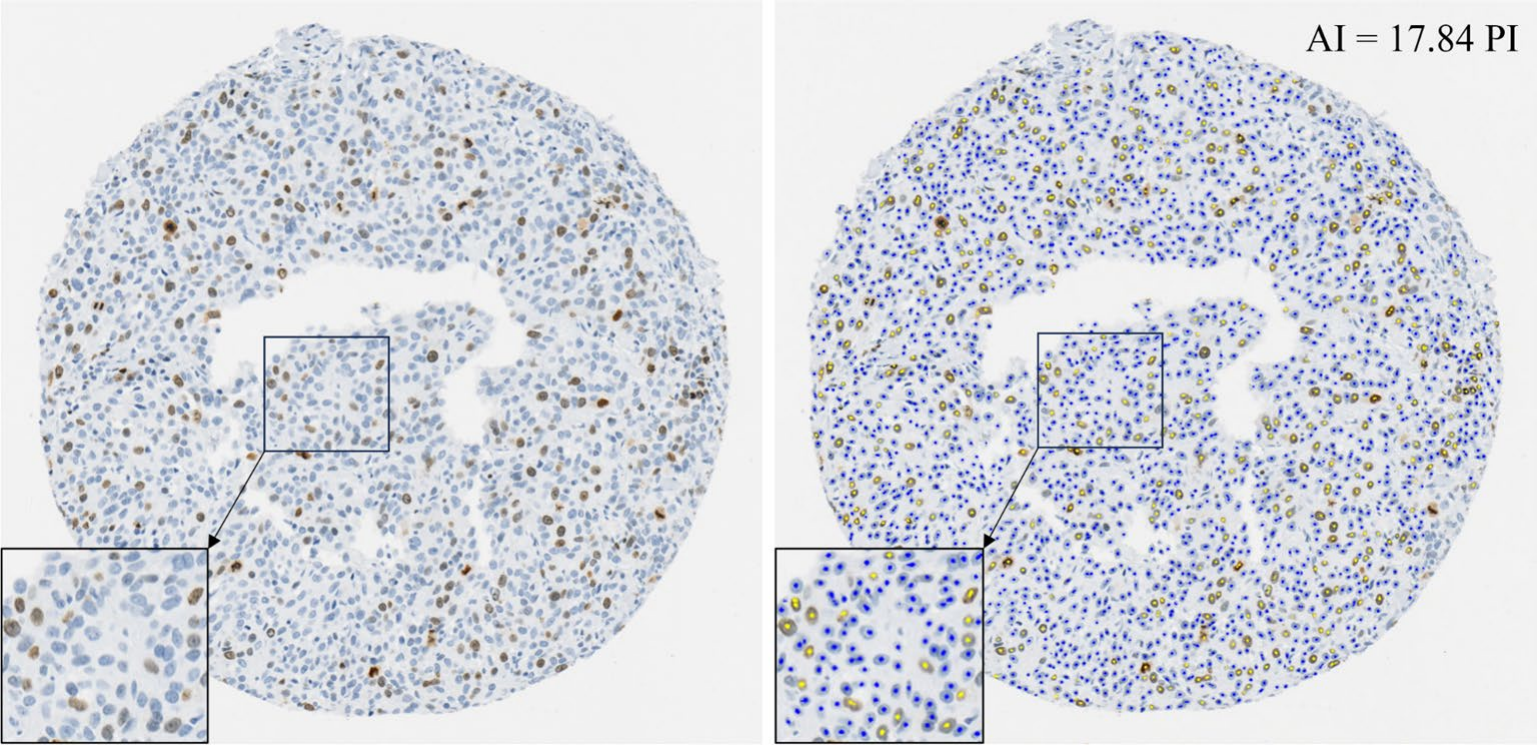
Examples of TMA with no AI aid (left) and TMA with AI tool overlay and calculated proliferation index (PI) (right). The TMA shown is case 7.

For a given image, the AI tool generates an automated Ki-67 positive and Ki-67 negative overlay (Fig. 1), providing an accessible visual interpretation along with the automated PI calculation.

The generalizability of UV-Net was previously validated on multi-institutional datasets from 5 institutions^18^, including WSIs and TMAs from breast cancer images. UV-Net consistently outperformed other architectures across all image variations, registering an average F1-score of 0.83 on expertly annotated data. In comparison, alternative architectures achieved scores between 0.74 and 0.79.

The images on which UV-Net was trained differed from those used in this study, originating from datasets with different scanners and institutions. None of the pathologists involved in this study participated in annotating the training or validation datasets.

### Study design

A cross-sectional study was performed using an anonymous, self-administered, and structured online survey developed using Qualtrics™, which included hyperlinks for viewing digitized TMAs on the cloud through PathcoreFlow™, a browser-based commercial image management solution and viewer for digital pathology^27^. The AI tool for Ki-67 scoring was integrated into PathcoreFlow™ using an Application Programming Interface. The tool provided an overlay of the Ki-67 positive and negative nuclei and calculated PI scores (Fig. 1). Participants were presented with a digital invasive breast cancer TMA stained for Ki-67 for each question and were asked to assign a Ki-67 score by entering a percentage value into Qualtrics™. Examples of questions with and without AI assistance are shown in Supplementary Figs. 1 and 2. Each Ki-67 TMA was reviewed by respondents twice—once without AI assistance and once with AI assistance—resulting in a total of 20 assessment questions. Participants were not explicitly told to use the AI, but rather to observe the AI results and estimate their PI score. They were instructed to compute the Ki-67 PI by counting individual cells with a denomination of 500 cells and to regard any brown staining beyond the background as positive, in line with current guidelines^8,9^. They were also guided to spend approximately the same time they would during standard procedures with no limit on the time for assessments. Each pathologist used a distinct viewer from a separate workstation. To minimize bias, the order of cases was randomized, ensuring that TMAs with AI assistance were not shown immediately before or after the same TMA without assistance. Additionally, the AI-assisted images were altered in orientation to look different from the unaided images. At the end of the study, participants were requested to provide their demographic information and respond to inquiries regarding their perspectives on AI.

### Study population

Participants were recruited through the professional networks of the authors between September and November 2022. Contact channels included pathology associations, local pathology residency programs, pathologist colleagues, and social media platforms (LinkedIn, Twitter). Eligible participants were trained pathologists with experience in Ki-67 PI scoring. The study included all participants who provided consent and identified themselves as pathology specialists. There were no limitations based on gender, age, or employment status, and only those who finished the study were considered, in total there were 116 completed responses. Spurious responses defined as outliers with large PI errors (more than 20% on a single response) were excluded from the analysis (N = 26 participants). Consequently, the main analysis included 90 respondents, all experienced in using digital pathology. Demographic characteristics are described in Supplemental Table 1. The participants' median age ranged from 40 to 49 years; however, the most common age group was 30 to 39 years, accounting for 34.4% of the respondents. While the median work experience falls within the 10 to 19 years range, the most prevalent work experience category is 0 to 9 years, representing 26.7% of the total. The majority of respondents are male, with many being retired clinical pathologists from North America. Among those currently working, most practice in academic health sciences centers.

### Ground truth scores

The ground truth Ki-67 PI scores for the 10 TMAs were determined using the gold standard manual counting method, where any brown staining above the background level was deemed positive, following current guidelines^8,9^. Each TMA was divided into five rows and five columns, creating 400 × 400 pixel tiles, and annotations were made in each region. Nuclei were annotated at the center of each cell, with tumor cells marked as Ki-67 positive if any discernible brown staining above the background was observed and the cell border was visible; otherwise, they were marked as Ki-67 negative. In cases of overlapping tumor cells, each cell was marked individually if its borders were discernible. An anatomical pathology resident (N.N.J.N.) performed the manual annotations, which were verified by a breast pathologist (S.D.). Ground truth PI scores were calculated from these manual annotations. The ground truth PI scores of the ten cases ranged from 7 to 28%.

### Statistical analysis

Statistical analyses were performed to assess the PI scoring error, inter-rater agreement, and TAT among pathologists when using the AI tool, compared to a standard clinical workflow (i.e., without AI). The experiment involved two groups: a control group where pathologists evaluated Ki-67 PI using standard clinical methods, and an experimental group where the same pathologists used the AI tool to assist with Ki-67 PI assessment on the same TMAs. For each participant, two PI estimations and TATs were obtained per TMA, resulting in 900 paired assessments (90 pathologists × 10 cases). For every assessment, several metrics were recorded, including the clinician-estimated raw PI score, the PI error (the absolute difference between the estimated and ground truth PI), and TAT, which denotes the time taken to score the TMA. The paired Wilcoxon signed-rank test^28^ was used to compare the differences between the two groups, with significance determined based on the median values of the paired differences. This test was chosen due to the non-normal distribution of the data, as indicated by the Shapiro–Wilk test. All statistical analyses were two-sided, with significance set at *p* < 0.05.

PI scores and PI errors were assessed with and without AI assistance, using continuous and binary values. PI scores and PI errors were first treated as continuous values and summarized by the mean and standard deviation. Box and bar plots were used to visually depict case-based and sub-demographic PI errors, respectively. PI scores and errors were additionally binarized and assessed using low-risk Ki-67 PI < 20%, and high-risk ≥ 20% stratification^12^.

The consistency of scoring among pathologists, with and without AI assistance, was examined using both continuous and binary metrics. For the continuous analysis, the Two-Way Random-Effects Model for single-rater consistency agreement was chosen to assess the inter-rater agreement using the Intraclass Correlation Coefficient (ICC)^29,30^. This model was selected since all cases were evaluated by all raters. The choice of the single-rater model stemmed from the clinical reliance on a singular clinician's decision for Ki-67 scores, rather than averaging scores from multiple clinicians^29^. The ICC between the pathologists’ PI scores and the ground truth PI was assessed twice: once with and once without AI assistance. Complementary to ICC, Krippendorff's α was calculated to measure inter-rater agreement and chosen for its adaptability in handling continuous data^31^. Bland–Altman and linear regression plots of the PI scores were incorporated to supplement the measure of inter-rater agreement, with parameters such as Pearson’s correlation coefficient, slope, offset, mean, and limits of agreement being considered. Using binarized PI scores (with scores ≥ 20% assigned a 1, and scores < 20% a 0), the percent agreement and Fleiss’ Kappa^32^ were calculated for both groups.

The TAT among pathologists was considered the time in seconds to perform the PI score estimation, starting from the moment they began examining the case to the point when the PI score was saved. TATs were summarized by the mean and standard deviation. Box and bar plots were used to visually depict case-based and sub-demographic TATs, respectively. Additionally, the percentage of time reduction computed by the time savings was determined by the formula: (total time saved/total time spent on conventional assessment) X 100%. Statistical analyses were performed using SPSS Version 28 (Armonk, NY, USA).

### Ethics approval

This research study has been reviewed by the Toronto Metropolitan University Research Ethics Board (REB 2022-154). Participants voluntarily consented to participate and to share contact information if they wanted to.

## Results

### Scoring accuracy

**Figure 2:**
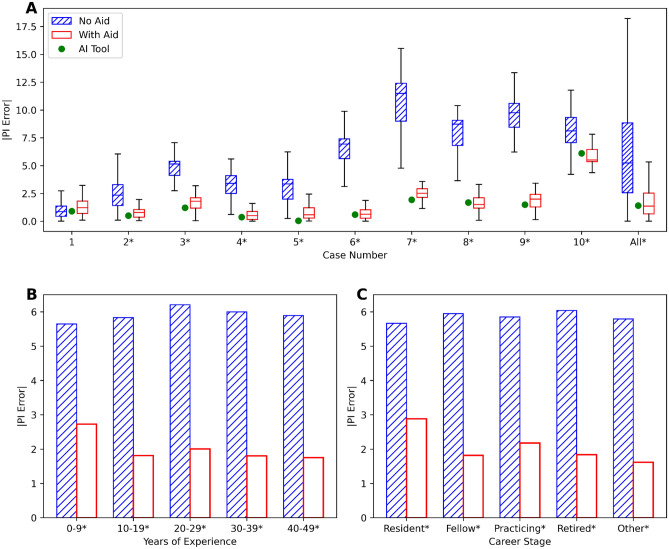
Graphs of absolute PI error. (**A**) Illustrates the absolute PI error vs. each case. (**B**) Displays the mean absolute PI error vs. years of experience. (**C**) Depicts the mean absolute PI error vs. career stages. Asterisk: statistical significance was found between pathologists and pathologists with AI using the paired Wilcoxon signed-rank test.

The respondents' PI scores and PI errors per case and within ranges are shown in Table 1. Responses including outliers are shown in Supplementary Table 2. The overall mean PI error was found to be 2.1 (2.2) using the AI tool, and 5.9 (4.0) without the AI (difference of − 3.8%, 95% CI: −4.10% to −3.51%, *p* < 0.001). The AI tool significantly improved the accuracy of PI scoring. The PI error was plotted per case (Fig. 2A) and for each PI interval (Supplementary Fig. 3). Cases 2 through 10 had significantly less error (*p* < 0.001), and both the < 20% and ≥ 20% PI ranges had statistically significant decreases in error with AI (*p* < 0.001). Furthermore, Fig. 2B, C, which display the PI error across various demographics, revealed that AI-aided scoring was superior across all pathologist age ranges and experience levels—indicating that despite variable background and training, AI improved PI accuracy for all groups of pathologists. Supplementary Fig. 4 shows that AI-aided scoring was statistically superior (*p* < 0.001) across all pathologist subdisciplines.

**Table 1:** PI scores, PI error and ground truth are shown as mean (SD) per case and within PI ranges.

Case	Ground truth	AI tool	AI tool error	PI scores			PI error		
				No aid	With aid	*p* value*	No aid	With aid	*p* value*
1	7.3	8.2	0.9	8.0 (4.4)	8.6 (1.3)	** < 0.001**	2.1 (3.9)	1.5 (1.1)	0.133
2	11.1	10.5	0.6	10.6 (4.3)	10.8 (1.4)	** < 0.001**	3.0 (3.1)	1.0 (1.0)	** < 0.001**
3	12.1	13.3	1.2	8.6 (3.9)	13.8 (1.9)	** < 0.001**	4.9 (1.8)	2.0 (1.6)	** < 0.001**
4	14.4	14.8	0.4	12.6 (3.5)	15.0 (2.3)	** < 0.001**	3.5 (1.8)	1.1 (2.1)	** < 0.001**
5	16.2	16.2	0.0	14.3 (3.6)	16.6 (1.9)	** < 0.001**	3.4 (2.9)	1.1 (1.5)	** < 0.001**
6	16.9	16.3	0.6	11.1 (4.0)	16.8 (1.5)	** < 0.001**	6.5 (2.5)	1.0 (1.2)	** < 0.001**
7	19.8	17.8	2.0	28.6 (6.0)	18.0 (3.6)	** < 0.001**	9.9 (3.9)	3.0 (2.6)	** < 0.001**
8	23.7	22.0	1.7	17.3 (5.5)	22.0 (2.2)	** < 0.001**	7.9 (2.9)	2.0 (1.9)	** < 0.001**
9	28.2	29.7	1.5	19.8 (4.7)	30.3 (2.7)	** < 0.001**	9.2 (2.9)	2.5 (2.3)	** < 0.001**
10	27.8	33.9	6.1	19.6 (4.1)	32.3 (3.8)	** < 0.001**	8.5 (3.4)	5.6 (1.8)	** < 0.001**
All	17.7	18.3	0.6	15.0 (7.5)	18.4 (7.7)	** < 0.001**	5.9 (4.0)	2.1 (2.2)	** < 0.001**
< 20%	14.0	13.7	0.3	19.6 (4.1)	14.2 (3.8)	** < 0.001**	4.8 (3.8)	1.5 (1.8)	** < 0.001**
≥ 20%	26.6	28.5	1.9	18.9 (4.9)	28.2 (5.4)	** < 0.001**	8.5 (3.1)	3.4 (2.6)	** < 0.001**

*The p values were computed for paired comparisons between pathologists and pathologists with AI with the paired Wilcoxon signed-rank test.

Significant values are in bold.

The AI tool demonstrated high accuracy in the study, with a mean PI error rate of 0.6%, which ranged from 0.0 to 6.1%, as shown in Table 1.

To quantify the increase of PI estimation accuracy when pathologists used the AI tool, Supplementary Fig. 5 shows the difference in PI error for each case. This difference is calculated as the PI error for the estimated PI score with AI assistance minus the error without AI assistance, highlighting the extent to which the AI tool reduces error rates. Most pathologists experienced increased accuracy with the AI tool, as indicated by positive differences seen in Supplementary Fig. 5.

### Inter-rater agreement

**Figure 3:**
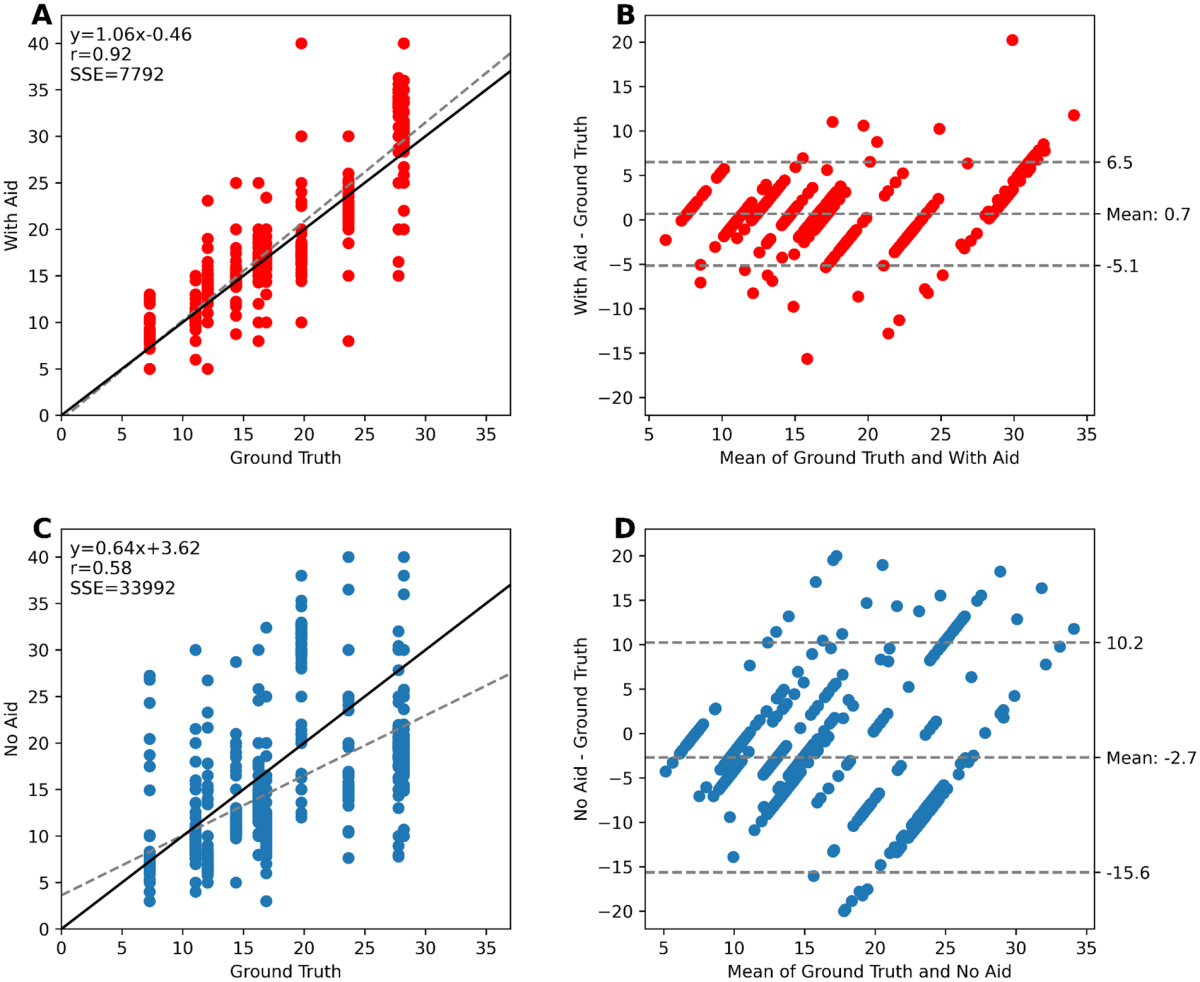
(**A**) Linear Regression of pathologists’ scores with AI assistance. (**B**) Bland–Altman of pathologists’ scores with AI assistance. (**C**) Linear regression of pathologists’ scores without AI assistance. (**D**) Bland–Altman of pathologists’ scores without AI assistance.

AI assistance led to a significant improvement in inter-observer reproducibility (with AI assistance: ICC = 0.92 [95% CI 0.85–0.98], Krippendorff’s α = 0.89 [95% CI 0.71–0.92], without AI assistance: ICC = 0.70 [95% CI 0.52–0.89], Krippendorff’s α = 0.65 [95% CI 0.41–0.72]). These statistics are visually depicted in Supplementary Fig. 6. Bland–Altman analyses (Fig. 3B, D) revealed that pathologists with AI assistance exhibited less bias (mean of 0.7 vs. − 2.7) and tighter limits of agreement (6.5 to − 5.1 vs. 10.2 to − 15.6) compared to the ground truth scores. Linear regression models (Fig. 3A, C) further support the notion that AI assistance improves inter-rater agreement (with AI assistance: y = 1.06x − 0.46, r = 0.92, SSE = 7792; without AI assistance: y = 0.64x + 3.62, r = 0.58, SSE = 33,992).

After binarizing the pathologists' responses, with scores ≥ 20% assigned as 1 and scores < 20% as 0, the Fleiss’ Kappa values showed better agreement with AI assistance (with AI assistance: 0.86 [95% CI 0.85–0.86]; without AI assistance: 0.40 [95% CI 0.40–0.41]). Table 2 shows that agreement levels are increased for every case when using AI, with some cases achieving 100% agreement.

**Table 2:** Average percent agreement for each of the 10 cases and cases above and below a 20% PI threshold.

Average percent agreement				
Case	Ground truth (PI)	Tool (PI)	No aid (%)	With aid (%)
1	7.3	8.2	95.6	100.0
2	11.1	10.5	94.4	100.0
3	12.1	13.3	96.7	98.9
4	14.4	14.8	92.2	95.6
5	16.2	16.2	91.1	96.7
6	16.9	16.3	95.6	94.4
7	19.8	17.8	88.9*	86.7
8	23.7	22.0	76.7*	96.7
9	28.2	29.7	67.8*	100.0
10	27.8	33.9	56.7	97.8
1–7	< 20%		82.4	96.0
8–10	≥ 20%		73.2*	98.1

The * highlights situations where the consensus misaligns with the established ground truth—meaning pathologists agree on a PI score that's > 20%, but the actual score was < 20%. For example, in case 7, most raters agreed that the TMA had a PI above 20%, but the ground truth indicates it's below 20% PI.

### Turnaround time

**Figure 4:**
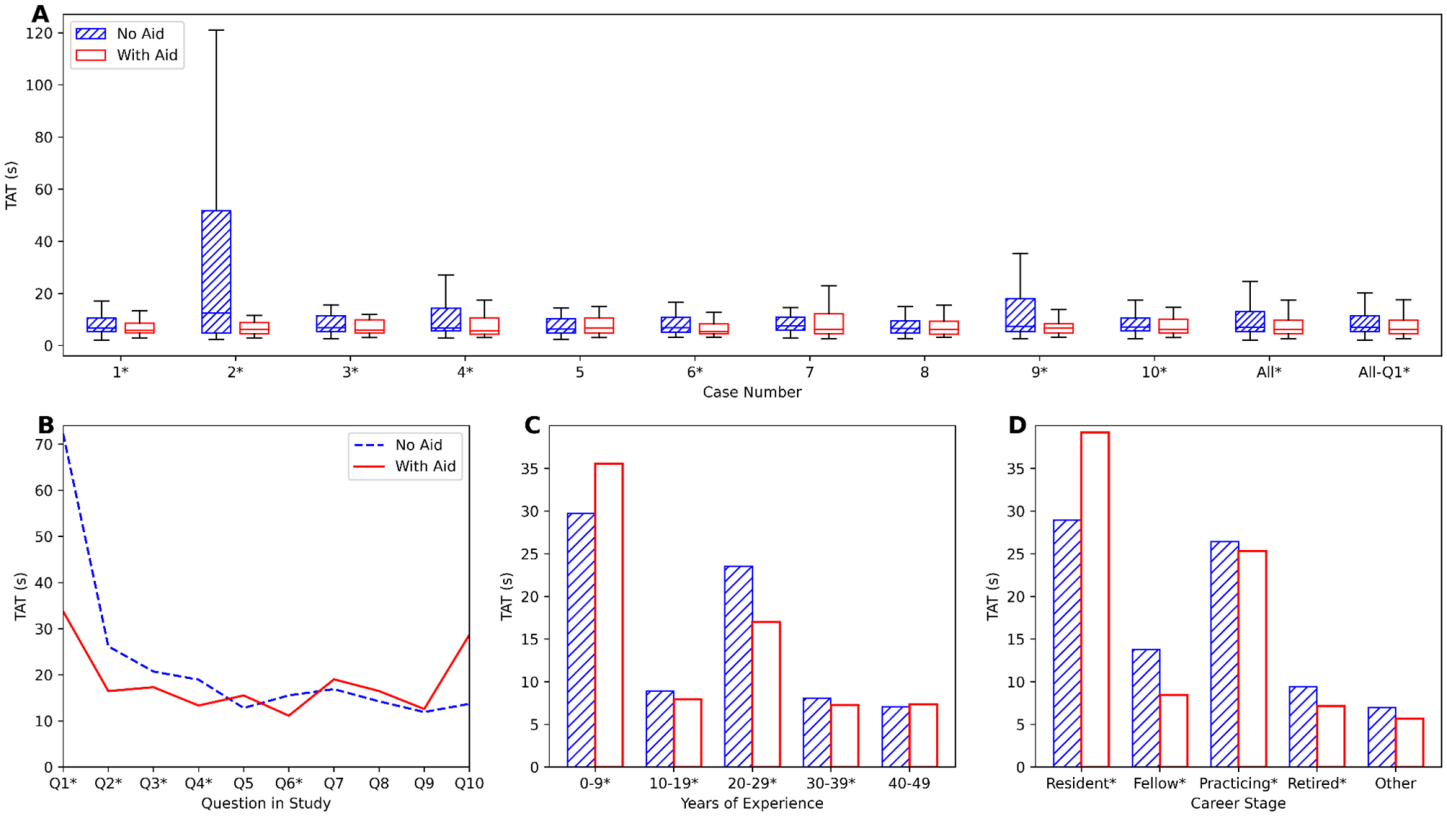
Graphs of TATs displayed in seconds. (**A**) Illustrates the absolute TAT vs. each case, the average of all cases (All), and the average of all cases excluding Question 1 (All-Q1). (**B**) Represents the TAT vs. the sequential question pairs in the study. (**C**) Displays the mean TAT vs. years of experience. (**D**) Depicts the mean TAT vs. career stages. Asterisk: Statistical significance was found between pathologists and pathologists with AI using the paired Wilcoxon signed-rank test.

A visual depiction of TATs for each case is provided in Fig. 4A. Table 3 displays the mean response time, standard deviation, and time saved for each TMA case for PI scoring with and without the AI aid.

**Table 3:** TAT expressed in seconds as mean (SD).

Case	No aid (s)	With aid (s)	With aid—no aid (s)	*p* value*
1	15.5 (34.3)	12.8 (28.6)	**−2.7**	**0.02**
2	71.7 (146.3)	15.3 (30.1)	**−56.4**	** < 0.001**
3	16.6 (25.6)	17.6 (31.2)	+ 1.0	0.08
4	22.2 (37.0)	18.6 (49.5)	**−3.6**	**0.003**
5	13.5 (23.1)	14.2 (23.4)	+ 0.7	0.35
6	19.2 (44.4)	25.0 (99.8)	**+ 5.8**	**0.004**
7	14.9 (23.4)	36.4 (70.8)	+ 21.5	0.58
8	12.0 (18.8)	17.0 (34.6)	+ 5.0	0.81
9	27.4 (57.1)	12.7 (19.6)	**−14.7**	** < 0.001**
10	20.1 (38.4)	16.2 (50.8)	**−3.9**	**0.006**
All	23.3 (59.4)	18.6 (50.1)	**−4.6**	** < 0.001**
All—Q1	18.3 (48.6)	16.8 (36.9)	**−1.5**	** < 0.001**

*The p values were computed for paired comparisons between pathologists and pathologists with AI with the paired Wilcoxon signed-rank test.

Significant values are in bold.

Without AI assistance, pathologists required an average of 23.3 s to assess each TMA, with a median time of 7.5 s and an interquartile range (IQR) of 5.5 to 16.2 s. AI assistance led to a statistically significant increase (*p* < 0.001) in efficiency where the average TAT per TMA reduced to 18.6 s, a median time of 6.4 s and a narrower IQR from 4.6 to 12.1 s.

Figure 4B illustrates the TAT for each question, showing the progression of TAT across cases as they were presented to the pathologists. Due to initially high response times, likely caused by participants acclimating to the software and study setup, question 1 (Case 2 without aid and Case 7 with aid) was excluded from further analyses.

For evaluations without AI, pathologists averaged 18.3 s per TMA, with a median time of 7.2 s and an IQR of 5.5 to 14.0 s. With AI support, the average TAT per TMA decreased to 16.8 s, the median time was 6.4 s, and the IQR narrowed to 4.7 to 11.6 s. The reduction in TAT was statistically significant among pathologists with experience ranging from 10 to 39 years (Fig. 4C) (*p* < 0.001), and for pathology fellows, practicing and retired pathologists (Fig. 4D) (*p* < 0.001). Supplemental Fig. 7shows the mean TAT with and without aid for various disciplines, where roles such as clinical and forensic pathologists were statistically faster (*p* < 0.001).

AI assistance resulted in an average reduction of 1.5 s per TMA [95% CI, −2.4 to −0.6 s, *p* < 0.001]. Supplementary Fig. 8 displays a histogram of the distribution of the total percentage of time saved, calculated using the formula: (total time saved/total time spent on conventional assessment) × 100%. The mean percentage saving was 9.4%, with a median of 11.9%.

### Pathologists’ opinions

**Figure 5:**
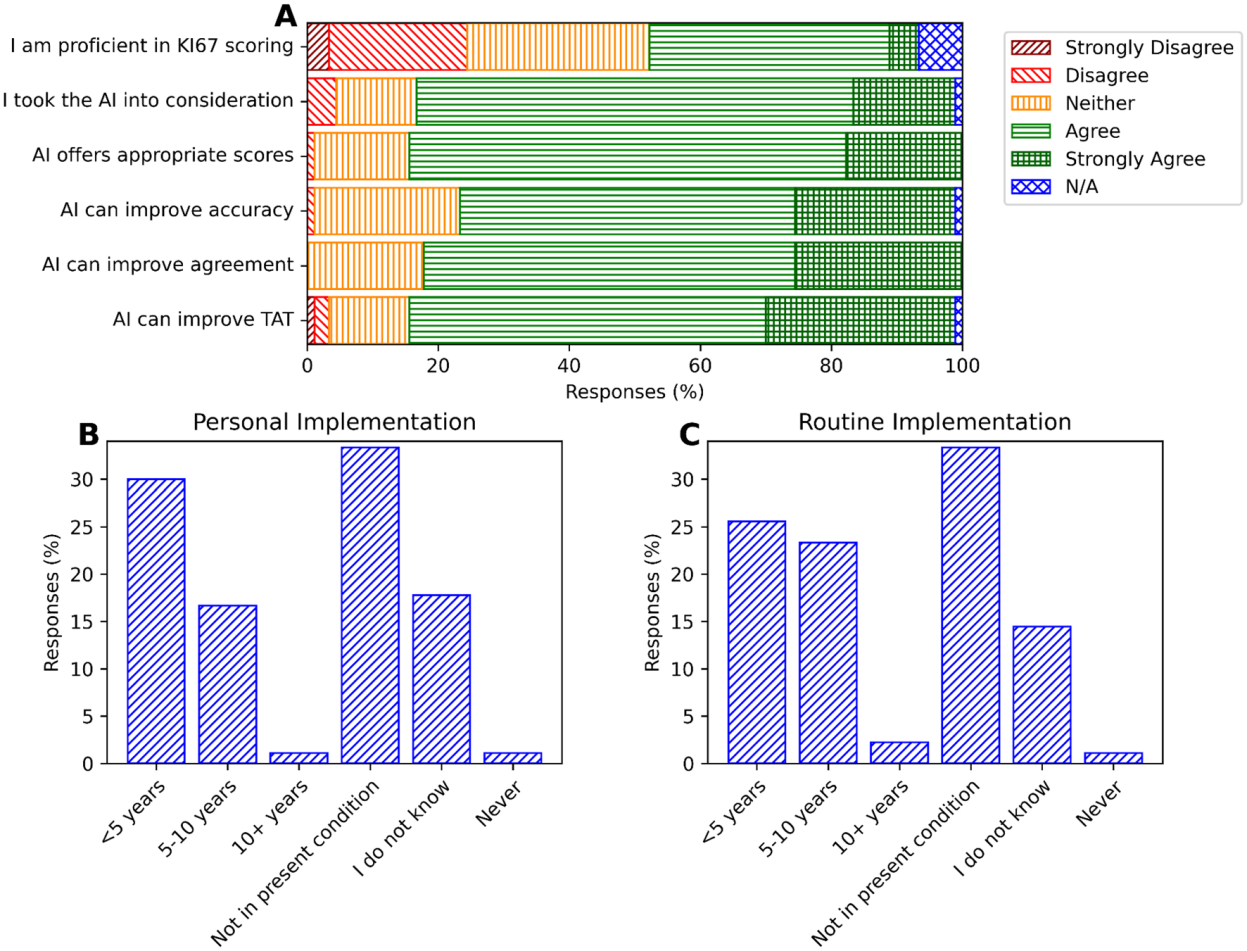
(**A**) Pathologists’ opinions on AI for Ki-67 assessments. (**B**) Pathologists’ opinions on their personal implementation timeline of AI into Ki-67 assessments. (**C**) Pathologists’ opinions on the routine implementation timeline of AI into Ki-67 assessments.

Pathologists’ opinions on the use of AI for Ki-67 assessment in breast cancer are summarized in Fig. 5. The majority of respondents considered the AI tool's suggestion, found it to be appropriate and agreed that this AI tool could improve accuracy, inter-rater agreement and TAT for Ki-67 assessments (Fig. 5A). Many respondents also agreed that they would personally implement and agree with the routine implementation of AI aid for Ki-67 assessments within the next decade (Fig. 5B, C).

## Discussion

Ki-67 serves as a crucial indicator for predicting cancer recurrence and survival among early-stage high-risk breast cancer patients^1,2^. It informs decisions regarding adjuvant chemotherapy^12^ and radiation therapy opt-out for Luminal A breast cancer patients^13^. These clinical decisions often rely on PI scores between 5 and 30%; however, this range exhibits significant scoring variability among experts, making standardization and clinical application challenging^8,9,12^. This inconsistency, combined with long assessment times using the current Ki-67 scoring system, has limited the broader clinical application of Ki-67 and resultantly, has not yet been integrated into all clinical workflows^16^. AI technologies are being proposed to improve Ki-67 scoring accuracy, inter-rater agreement, and TAT. This study explores the influence of AI in these three areas by recruiting 90 pathologists to examine ten breast cancer TMAs with PIs in the range of 7 to 28%.

Two previous studies aimed to quantify PI accuracy with and without AI^19,23^. One study demonstrated that AI-enhanced microscopes improved invasive breast cancer assessment accuracy^23^. They had 30 pathologists use an AI microscope to evaluate 100 invasive ductal carcinoma IHC-stained whole slide images (WSIs), which provided tumor delineations, and cell annotations. AI use resulted in a mean PI error reduction from 9.60 to 4.53. A similar study was conducted^19^, where eight pathologists assessed 200 regions of interest using an AI tool. Pathologists identified hotspots on WSIs, after which the AI tool provided cell annotations for the clinician's review. The study found that this method significantly improved the accuracy of Ki-67 PI compared to traditional scoring (14.9 error without AI vs. 6.9 error with AI).

Similarly, this study found that using AI assistance for PI scoring significantly (*p* < 0.001) improved pathologists’ accuracy, reducing both the PI error and its standard deviation across various demographics, including years of experience and specialties. This indicates that AI assistance leads to higher PI accuracy across all levels of pathologists' training, enabling professionals at every career stage to deliver more precise PI scores in the range critical for clinical decision-making. This improvement may help bridge experience gaps and is critical for PI scoring standardization. An underestimation trend, previously reported by^33^, was also noted in this study, as shown by the PI correlation and Bland–Altman analysis (Fig. 3). However, scoring with the support of AI improved PI accuracy for all cases and corrected this underestimation bias. This is exemplified by the scoring near the 20% cutoff, which simulates a clinical decision threshold. In conventional assessments, many pathologists select the incorrect range (≥ 20% or < 20%), particularly for TMAs 7, 8, and 9, with ground truths of 19.8, 23.7, and 28.2, respectively. For instance, TMA 8 had 76.7% of respondents incorrectly estimated the score as < 20%. Errors like these would result in incorrect therapy decisions and poor patient outcomes. Fortunately, with AI assistance, the percentage of pathologists agreeing with the ground truth greatly improved, providing a strong incentive for the clinical use of AI tools in Ki-67 scoring. All cases showed a statistically significant PI error decrease with AI assistance, except for Case 1, with a ground truth PI score of 7.3% (p = 0.133). This exception could be attributed to fewer Ki-67 positive cells requiring counting, which likely simplified the scoring process.

In addition to accuracy, PI scoring agreement is critical to ensure that patients with similar disease phenotypes are delivered the proper therapeutic regimes. However, significant variability in Ki-67 scoring is widely recognized, even in established laboratories. A study led by^34^, found reproducibility among eight labs was only moderately reliable with contributing factors such as subjective judgements related to PI scoring and tumor region selection. Standardizing scoring methods becomes imperative, as transferring Ki-67 PIs and cutoffs between laboratories would compromise analytical validity. In another study by^35^, the variability in breast cancer biomarker assessments, including Ki-67, among pathology departments in Sweden was investigated. While positivity rates for HR and HER2 had low variability, there was substantial variation in Ki-67 scoring, where 66% of labs showed significant intra-laboratory variability. This variability could potentially affect the distribution of endocrine and HER2-targeted treatments, emphasizing the need for improved scoring methods to ensure consistent and dependable clinical decision-making. The study by^23^, aimed to improve Ki67 scoring concordance with their AI-empowered microscope. They found a higher ICC of 0.930 (95% CI: 0.91–0.95) with AI, compared to 0.827 (95% CI: 0.79–0.87) without AI. Similarly^22^, aimed to quantify the inter-rater agreement for WSIs with AI assistance across various clinical settings. The AI tool evaluated 72 Ki-67 breast cancer slides by annotating Ki-67 cells and providing PI scores. Ten pathologists from eight institutes reviewed the tool and input their potentially differing PI scores. When the scores were categorized using a PI cutoff of 20%, there was an 87.6% agreement between traditional and AI-assisted methods. Results also revealed a Krippendorff's α of 0.69 in conventional eyeballing quantification and 0.72 with AI assistance indicative of increased inter-rater agreement, however, these findings were not significant.

In this study, we evaluated the scoring agreement with and without AI across 90 pathologists, representing one of the largest cohorts analyzed for this task. It was found that over the critical PI range of 7 to 28%, AI improved the inter-rater agreement, with superior ICC, Krippendorff’s α and Fleiss’ Kappa values compared to conventional assessments and higher correlation of PI estimates with the ground truth PI score. Additionally, there was a decrease in offset and variability, as shown in Fig. 3. These agreement metrics align with findings from earlier studies^22,23^ and signify that AI tools can standardize Ki-67 scoring, enhance reproducibility and reduce the subjective differences seen with conventional assessments. Therefore, using an AI tool for Ki-67 scoring could lead to more robust assessments and consistent therapeutic decisions.

AI applications have predominantly focused on automating the laborious tasks for pathologists, thereby freeing up time for high-level, critical decision-making, especially those related to more complex disease presentations^16–20,36^. Some research into AI support tools in this field has demonstrated a notable decrease in TAT for pathologists. For instance, a study led by^37^, which involved 20 pathologists analyzing 240 prostate biopsies, reported that an AI-based assistive tool significantly reduced TAT, with 13.5% less time spent on assisted reviews than on unassisted ones. Similarly, the study by^38^, demonstrated a statistical improvement (*p* < 0.05) in TATs when 24 raters counted breast mitotic figures in 140 high-power fields, with and without AI support, ultimately achieving a time saving of 27.8%. However, the study by^23^, reported a longer TAT using an AI-empowered microscope in their study, which involved 100 invasive ductal carcinoma WSIs and 30 pathologists (11.6 s without AI vs. 23.8 s with AI).

Our study found that AI support resulted in faster TATs (18.3 s without AI vs. 16.8 s with AI, *p* < 0.001), equating to a median time saving of 11.9%. Currently, our team only performs Ki-67 testing upon oncologists' requests, as routine Ki-67 assessment is not yet standard practice. This is partly due to the difficulties in standardizing Ki-67, compounded by pathologists' increasing workloads and concerns over burnout^39,40^. Pathologists' caseloads have grown in the past decade, from 109 to 116 annually in Canada and 92 to 132 in the U.S.^41^. With the Canadian Cancer Society expecting 29,400 breast cancer cases in 2023^42^, routine Ki-67 assessments would significantly increase workloads. Therefore, the implementation of AI tools in this context could alleviate workload pressures by offering substantial time savings and supporting the clinical application of this important biomarker.

The gold standard for assessing Ki-67 PI is manual counting^8,9^; however, due to the labor-intensive nature of this method, many pathologists often resort to rough visual estimations^43,44^. As indicated in Table 3 and Fig. 4, the shorter TATs suggest that respondents may have relied on visual estimations for Ki-67 scoring. Despite this, the TATs significantly improved (*p* < 0.001) when using AI. This improvement was evident among experienced pathologists; however, some encountered longer TATs after integrating AI, possibly due to unfamiliarity with the AI tool or digital pathology viewing software. Although participants received a brief orientation and two initial examples, the novelty of the tool might have posed a learning curve. Addressing this challenge involves integrating the tool into regular practice and providing comprehensive training before its use.

The perspectives of pathologists highlight a growing enthusiasm towards AI integration for Ki-67 evaluations for breast cancer. A significant 84% of participants agreed the AI’s recommendations were suitable for the task at hand. They recognized AI's ability to improve pathologists' accuracy (76%), enhance inter-rater consistency (82%), and reduce the TAT for Ki-67 evaluations (83%). Additionally, 49% expressed their intent to incorporate AI into their workflow, and 47% anticipated the routine implementation of AI within the next decade. An important observation is that many respondents who were hesitant about personally or routinely implementing AI in clinical practice were retired pathologists. In total, 83% of retired pathologists reported they would not currently implement AI personally or routinely, which is a stark contrast to only 15% of practicing pathologists who expressed the same reluctance. This positive outlook in the pathologist community supports the insights of this study and signals an increasing momentum for the widespread adoption of AI into digital pathology.

The strength of this research is highlighted by the extensive and diverse participation of 90 pathologists, which contributes to the study's generalizability in real-world clinical contexts. Adding to the study's credibility is the focus on Ki-67 values around the critical 20% threshold, which is used for adjuvant therapy decisions. Moreover, the AI nuclei overlay addresses the transparency concerns often associated with AI-generated scores, thus improving clarity and comprehensibility for users. The ongoing discussion around 'explainable AI' highlights the importance of transparency in AI tools' outputs, a crucial factor for their acceptance and adoption^45^. The outcomes of the study emphasize the positive outlook and readiness of pathologists to embrace AI in their workflow and serve to reinforce the growing need for the integration of AI into regular medical practice.

The study has its limitations, one of which includes the potential unintentional inclusion of non-pathologists. The survey required respondents to confirm their status as pathologists through agreement before beginning; however, due to confidentiality limitations, no further verification was possible. In some instances, pathologists' scores deviated from the ground truth by more than 20%, with PI errors reaching up to 50%. Such large errors would render any PI score diagnostically irrelevant, as the variance exceeds the clinical threshold of 20%. These errors might be attributed to input errors or a lack of experience in Ki-67 assessments. Consequently, we used this threshold to filter out potentially erroneous responses. In total, 26 participants who logged responses exceeding the 20% error threshold were subsequently excluded from the study. For completeness, Supplementary Table 2 discloses the PI scores and PI errors of all respondents, including outliers, where the data trends appear similar. The demographics of the study's participants reveal there was limited participation from currently practicing pathologists, representing 14.4% of respondents. This may be attributed to the time constraints faced by practicing pathologists. In future research, efforts will be made to include more practicing pathologists and to evaluate intra-observer variability. Additionally, while the survey provided specific guidelines for calculating the PI and applying Ki-67 positivity criteria, the accuracy and thoroughness of each pathologist's evaluations could not be verified. Lastly, the study deviated from standard practice by using TMAs instead of WSIs for Ki-67 clinical assessments. The rationale behind this choice was the expectation of more precise scoring with TMAs, as this eliminates the need to select high-power fields (a subjective process) and involves a lower number of cells to evaluate, leading to better consistency in visual estimations. Future research should focus on evaluating the accuracy achieved with AI assistance in identifying regions of interest and analyzing WSIs. This should also incorporate a broader range of cases and a wider PI range. Prospective studies involving solely practicing breast pathologists could also yield valuable insights into the real-world application of the AI tool and its impact on clinical decision-making.

In conclusion, this study provides early insights into the potential of an AI tool in improving the accuracy, inter-rater agreement, and workflow efficiency of Ki-67 assessment in breast cancer. As AI tools become more widely adopted, ongoing evaluation and refinement will be essential to fully realize its potential and optimize patient care. Such tools are critical for robustly analyzing large datasets and effectively determining PI thresholds for treatment decisions.

### Supplementary Information

Supplementary Information.

## Data Availability

The datasets used and/or analyzed during the current study are available from the corresponding author upon reasonable request.
