# Supplementary material for: AI improves accuracy, agreement and efficiency of pathologists for Ki67 assessments in breast cancer
Source: Sci Rep. 2024 Jan 13;14:1283. doi: 10.1038/s41598-024-51723-2 (PMC10787826; doi:10.1038/s41598-024-51723-2)
Supplement: Supplementary file 1 — Supplementary Information. [file 41598_2024_51723_MOESM1_ESM.docx]

*
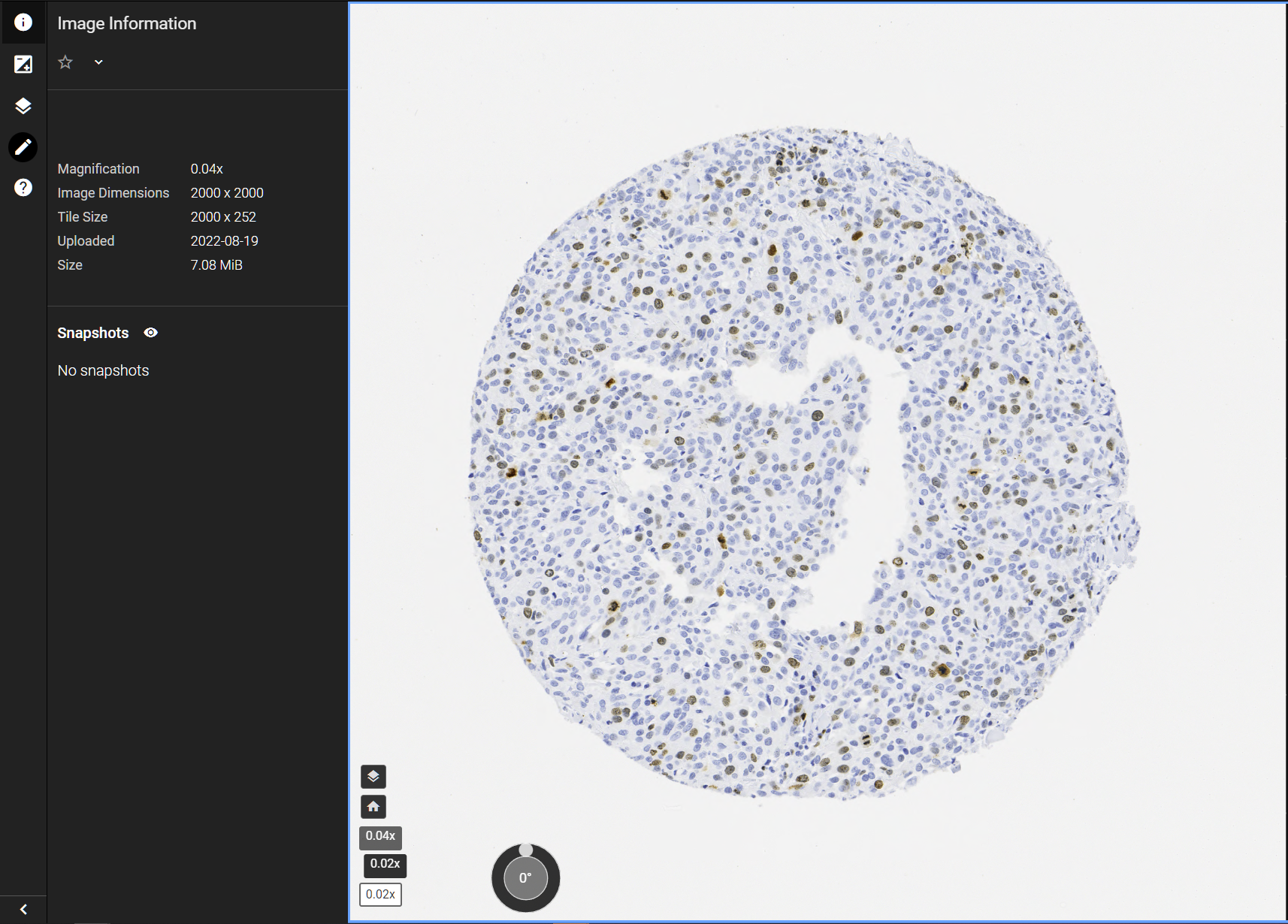
*

*Supplementary Figure 1. Sample Ki-67 scoring question without AI integration showing the PathcoreFlow^TM^ Digital Pathology Image Viewing and Management Software.*

*
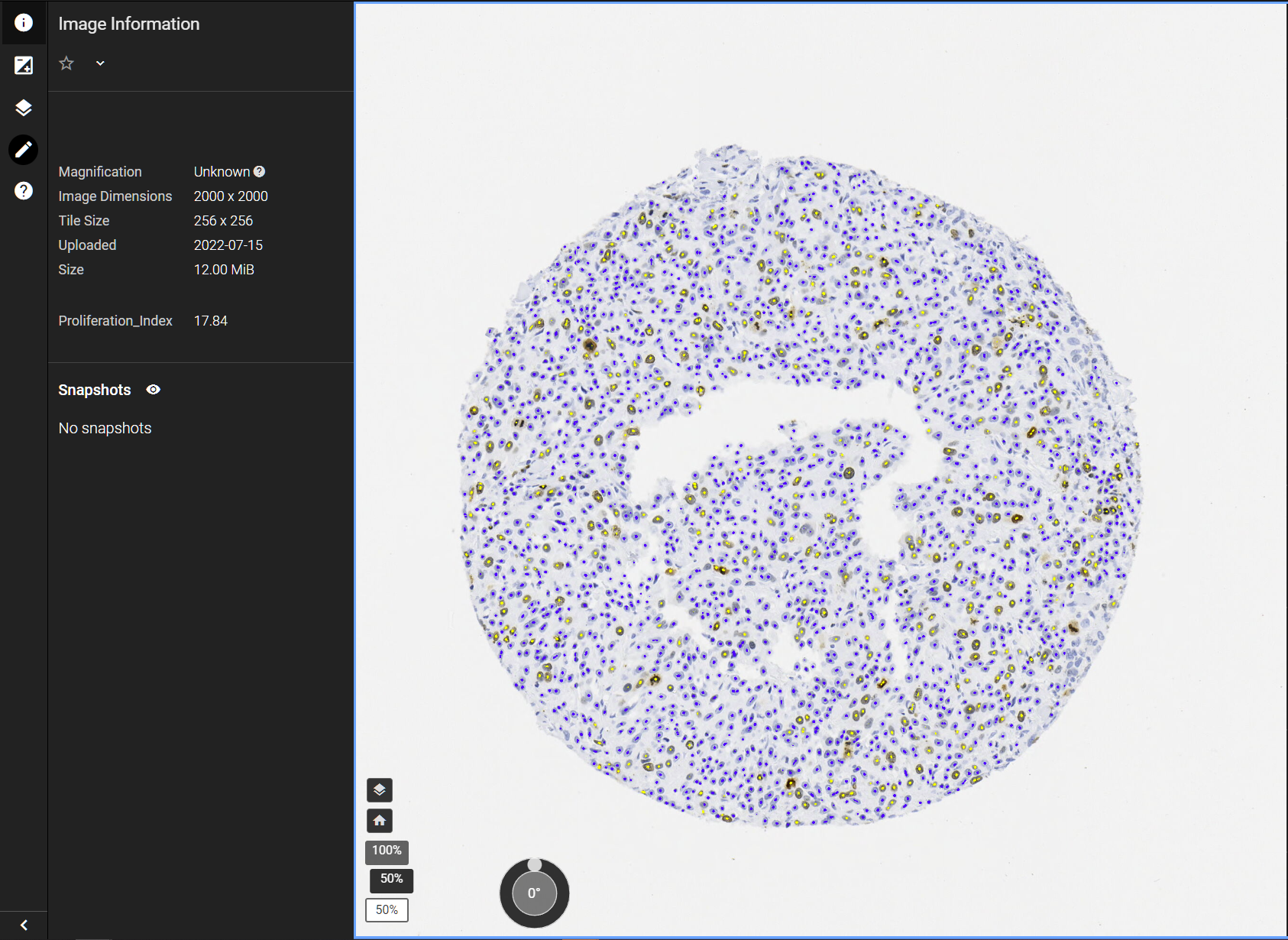
*

*Supplementary Figure 2. Sample Ki-67 scoring question with AI integration showing the PathcoreFlow^TM^ Digital Pathology Image Viewing and Management Software. Auto-generated PI of 17.84 and nuclei overlay are shown.*

Supplementary Table 1. Demographic characteristics of respondents (n=90). Abbreviations: Academic Health Sciences Center (AHSC). Current Workplace Setting has 95 responses because the question was ‘select all that apply’.

| **Variable** | **Number (%)** | **Variable** | **Number (%)** |
| --- | --- | --- | --- |
| **Age** | | **Pathology Discipline** | |
| 20-29 | 10 (11.1%) | Clinical Pathology | 29 (32.2%) |
| 30-39 | 31 (34.4%) | Anatomical Pathology | 19 (21.1%) |
| 40-49 | 13 (14.4%) | Other | 12 (13.3%) |
| 50-59 | 12 (13.3%) | Neuropathology | 9 (10.0%) |
| 60-69 | 24 (26.7%) | Pediatric Pathology | 9 (10.0%) |
| **Stage of Career** | | Microbiology | 6 (6.7%) |
| Pathology Resident | 19 (21.1%) | Hematologic Pathology | 3 (3.3%) |
| Pathology Fellow | 20 (22.2%) | Forensic Pathology | 3 (3.3%) |
| Practicing Pathologist | 13 (14.4%) | **Current Workplace Setting** | |
| Retired Pathologist | 31 (34.4%) | Not Currently Working | 26 (28.9%) |
| Other | 7 (7.8%) | AHSC | 24 (26.7%) |
| **Gender** | | Community Hospital | 16 (17.8%) |
| Male | 60 (66.7%) | Diagnostic Clinic | 13 (14.4%) |
| Female | 28 (31.1%) | Other Hospital | 9 (10.0%) |
| Other | 2 (2.2%) | Non-AHSC Teaching Hospital | 4 (4.4%) |
| **Years of Experience** | | University | 3 (3.3%) |
| 0-9 | 24 (26.7%) | **Region of Practice** | |
| 10-19 | 23 (25.6%) | North America | 84 (93.3%) |
| 20-29 | 16 (17.8%) | Europe | 4 (4.4%) |
| 30-39 | 23 (25.6%) | Asia | 2 (2.2%) |
| 40-49 | 4 (4.4%) |  |  |

Supplementary Table 2. PI scores, PI error and ground truth are shown per case and within PI ranges for all respondents (including outliers). *The p values were computed for paired comparisons between pathologists and pathologists with AI with the paired Wilcoxon signed rank test.

| **Case** | **Ground Truth** | **AI Tool** | **PI Scores** | | | **PI Error** | | |
| --- | --- | --- | --- | --- | --- | --- | --- | --- |
|  |  |  | **No Aid** | **With Aid** | ***p* Value*** | **No Aid** | **With Aid** | ***p* Value*** |
| 1 | 7.3 | 8.2 | 9.0 (6.4) | 8.9 (3.2) | **<0.001** | 3.4 (5.7) | 2.0 (3.0) | 0.930 |
| 2 | 11.1 | 10.5 | 11.7 (6.2) | 11.2 (3.3) | 0.094 | 3.8 (5.0) | 1.6 (2.9) | **<0.001** |
| 3 | 12.1 | 13.3 | 10.9 (8.1) | 13.8 (4.7) | **<0.001** | 6.1 (5.4) | 2.7 (4.2) | **<0.001** |
| 4 | 14.4 | 14.8 | 14.2 (7.8) | 15.4 (3.8) | **<0.001** | 5.0 (6.0) | 1.7 (3.6) | **<0.001** |
| 5 | 16.2 | 16.2 | 15.7 (5.5) | 17.0 (4.2) | **<0.001** | 4.2 (3.6) | 1.9 (3.8) | **<0.001** |
| 6 | 16.9 | 16.3 | 12.2 (5.9) | 16.7 (2.7) | **<0.001** | 6.6 (3.7) | 1.4 (2.3) | **<0.001** |
| 7 | 19.8 | 17.8 | 28.3 (8.0) | 18.8 (6.0) | **<0.001** | 10.0 (5.9) | 3.6 (4.9) | **<0.001** |
| 8 | 23.7 | 22.0 | 18.9 (8.2) | 22.3 (4.2) | **<0.001** | 8.4 (4.4) | 2.7 (3.5) | **<0.001** |
| 9 | 28.2 | 29.7 | 20.8 (7.8) | 31.3 (6.2) | **<0.001** | 9.6 (4.6) | 3.6 (6.0) | **<0.001** |
| 10 | 27.8 | 33.9 | 19.0 (5.9) | 32.0 (4.8) | **<0.001** | 9.4 (4.9) | 5.8 (2.6) | **<0.001** |
| All | 17.7 | 18.3 | 16.1 (8.9) | 18.8 (8.6) | **<0.001** | 6.7 (5.5) | 2.7 (4.0) | **<0.001** |
| <20% | 14.0 | 13.7 | 14.6 (9.1) | 14.6 (5.2) | **<0.001** | 4.8 (3.8) | 1.5 (1.8) | **<0.001** |
| ≥20% | 26.6 | 28.5 | 19.6 (7.4) | 28.6 (6.8) | **<0.001** | 8.5 (3.1) | 3.4 (2.6) | **<0.001** |

*
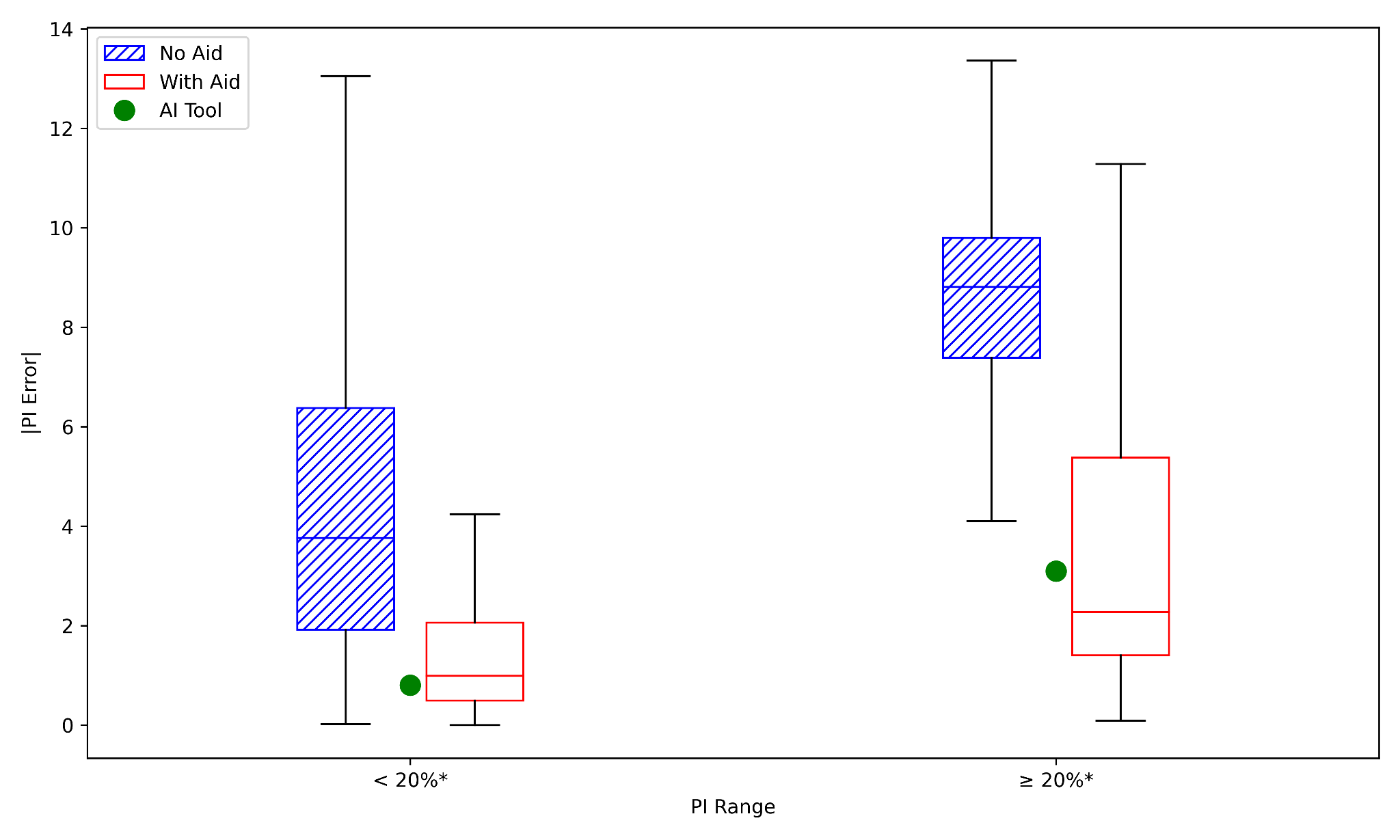
*

Supplementary Figure 3. The absolute PI error versus the PI Range. The intervals <20% and ≥20% PI demonstrate a statistically significant decrease in PI error when pathologists use AI assistance.

*
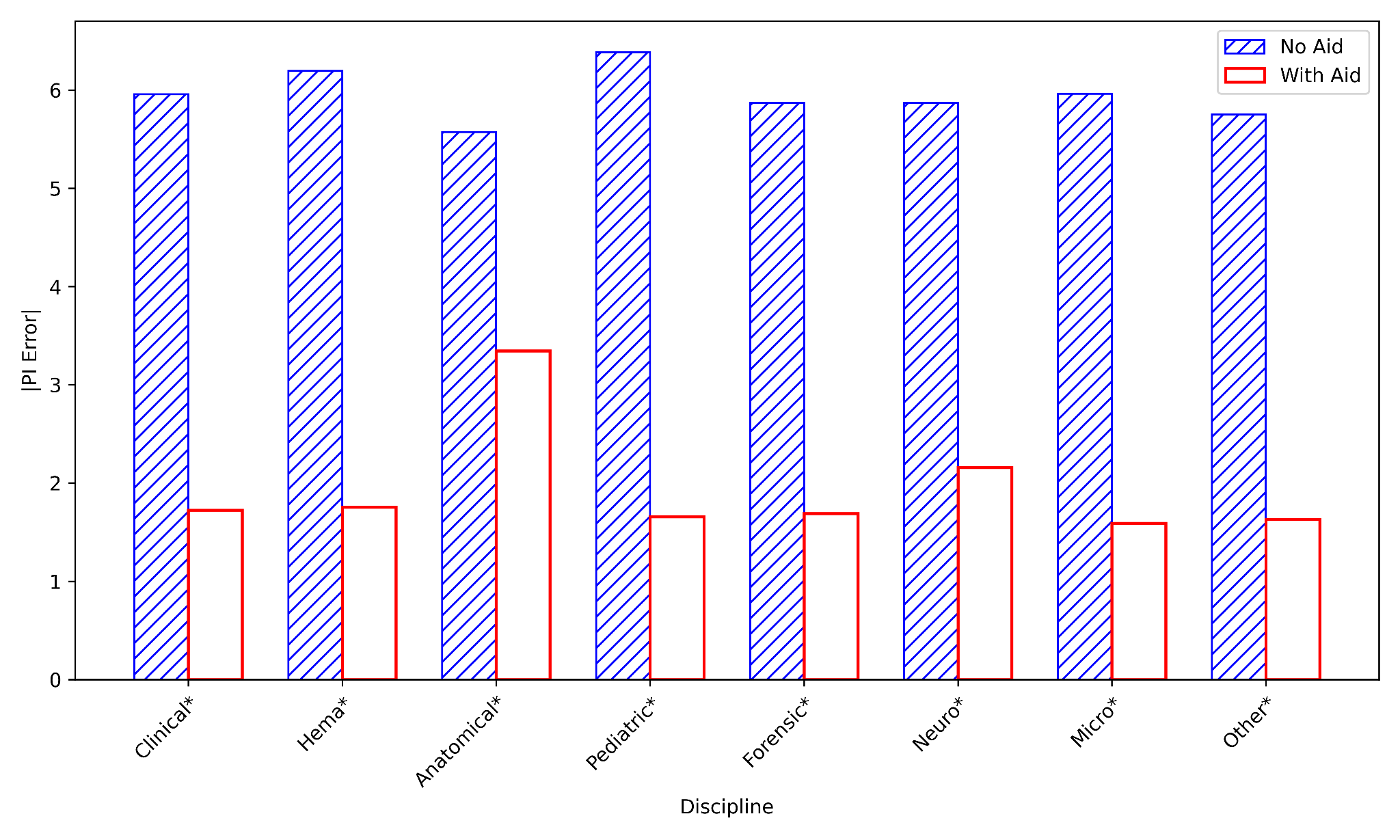
*Supplementary Figure 4. The mean PI error versus various pathology subdisciplines. Pathologists, irrespective of their specialization, were statistically more accurate with AI aid.


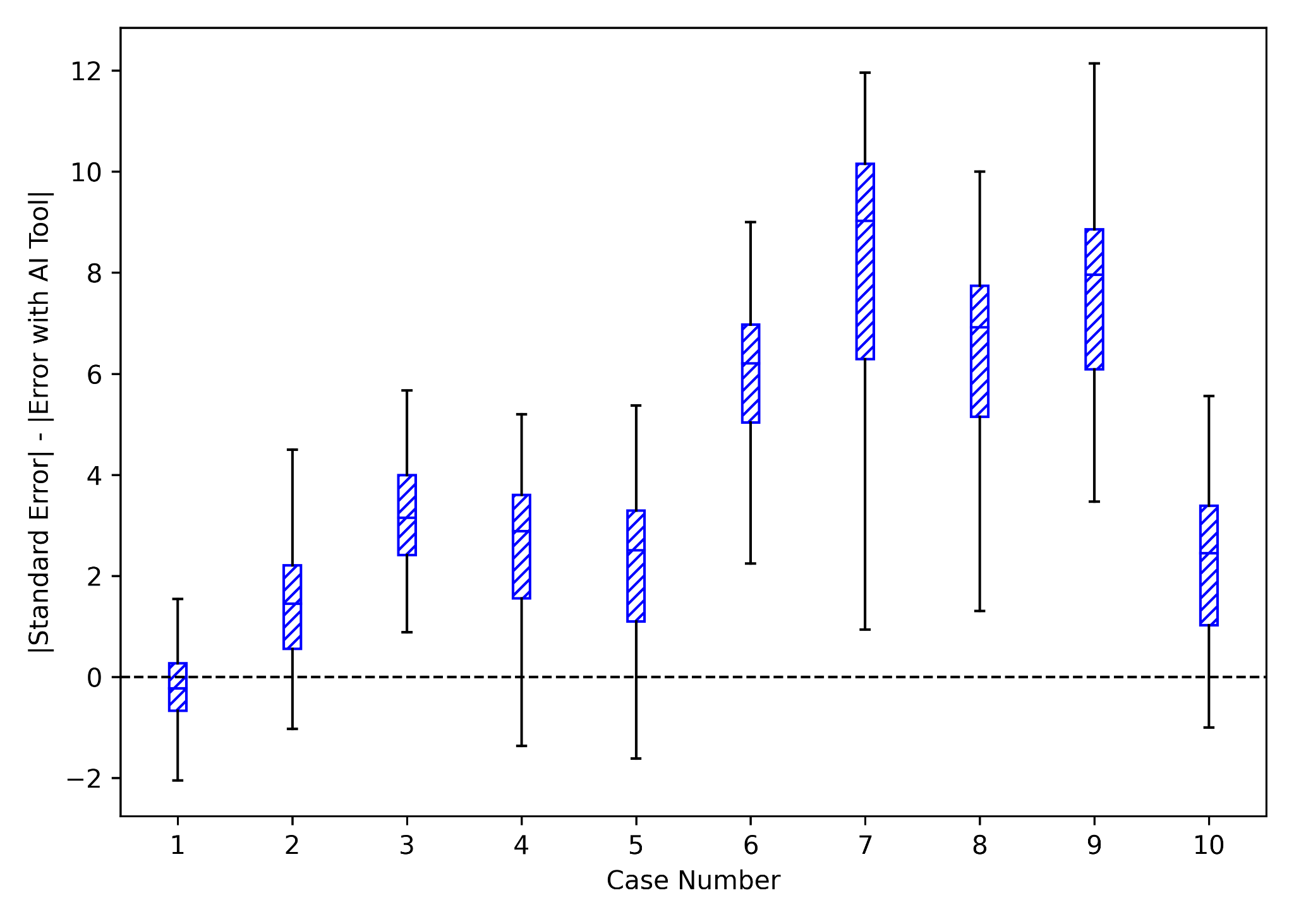


Supplementary Figure 5. This illustrates the variation in each pathologist's absolute error per case with AI assistance compared to the absolute error without AI. A positive difference value indicates increased accuracy when the pathologist utilized AI assistance, while a negative value means reduced accuracy when AI was used.

*
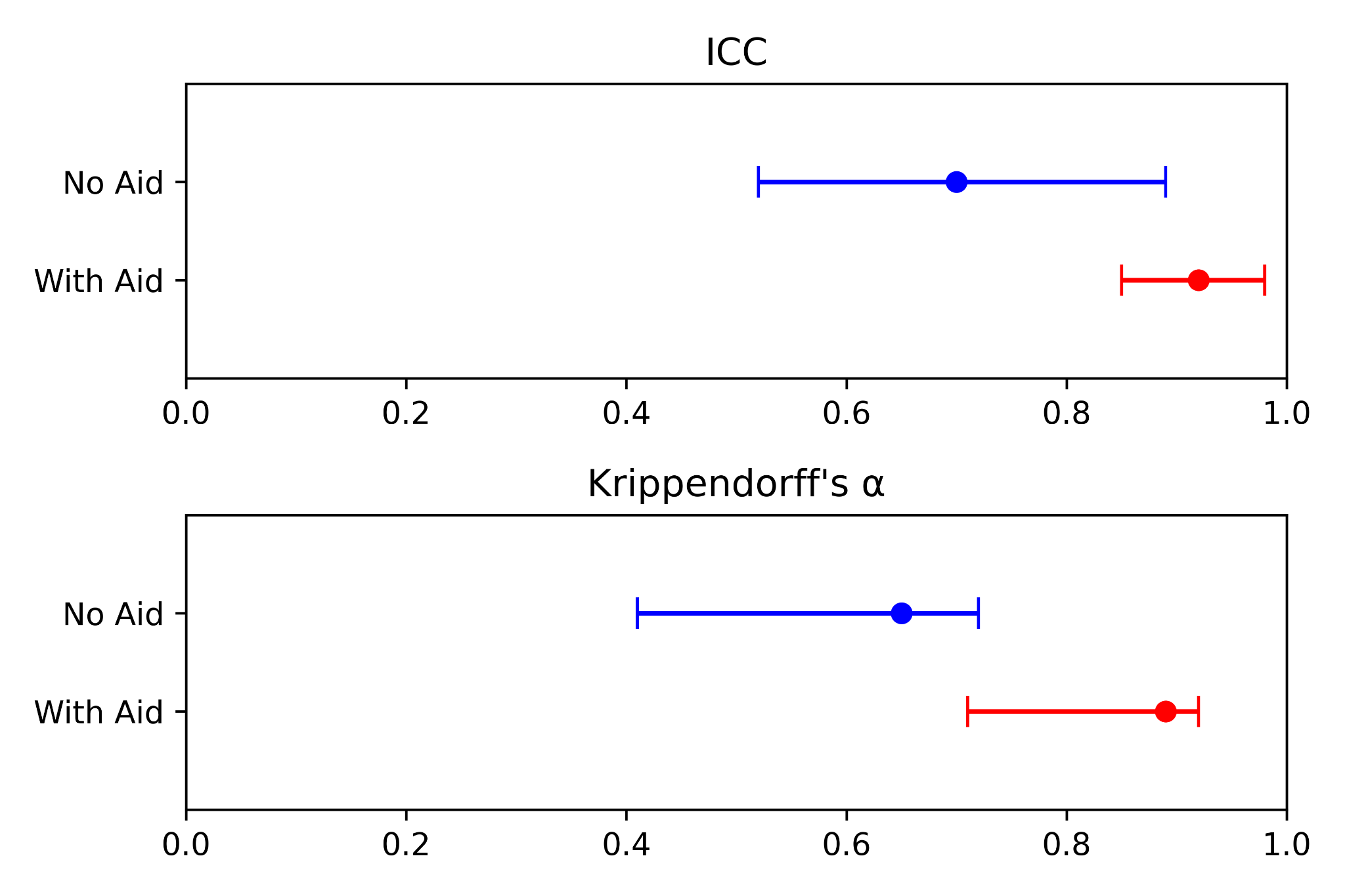
*

Supplementary Figure 6. ICC and Krippendorff’s α with and without AI assistance.

*
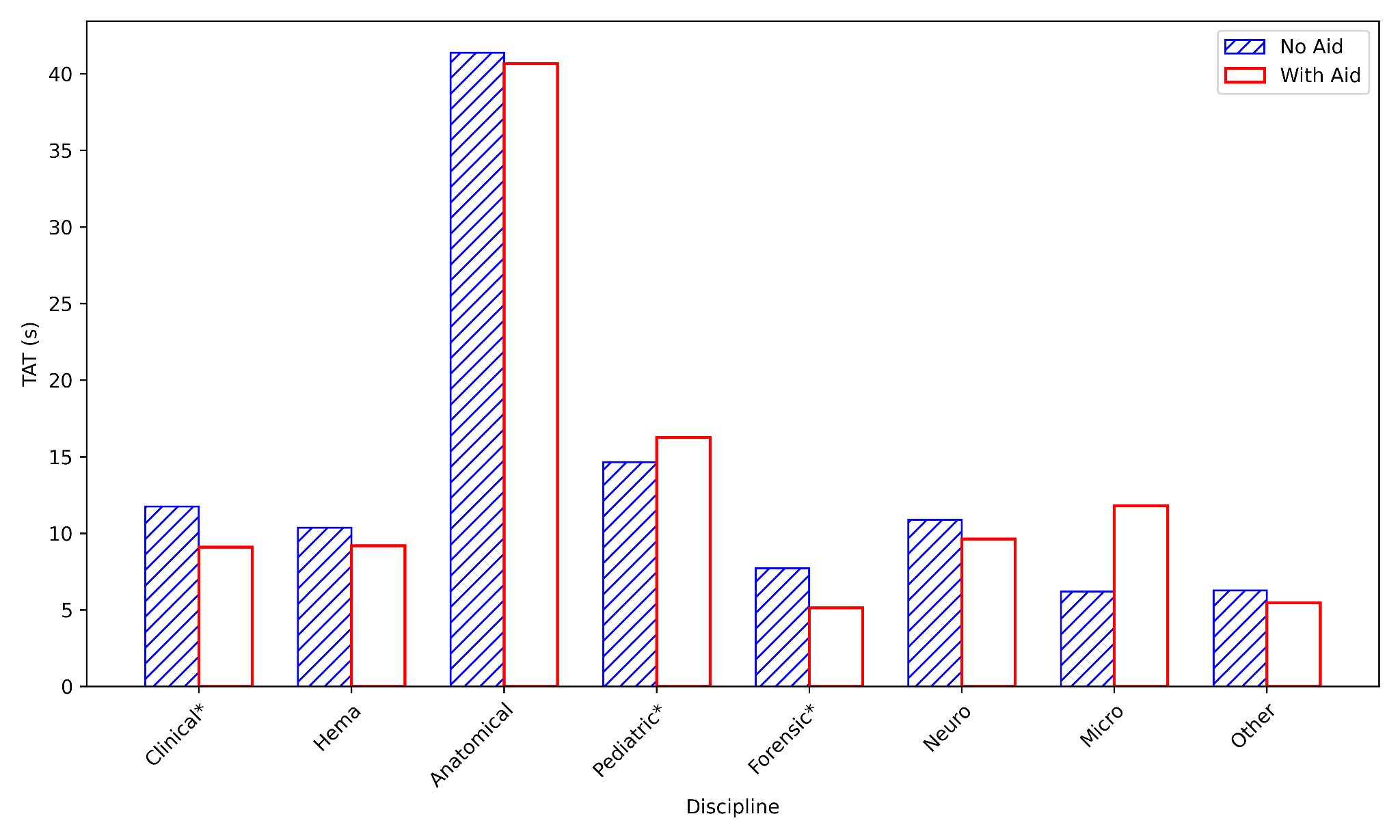
*Supplementary Figure 7. The mean turnaround time (TAT) versus various pathology subdisciplines. Pathologists with subspecialties clinical and forensic pathologists perform statistically faster with AI aid.


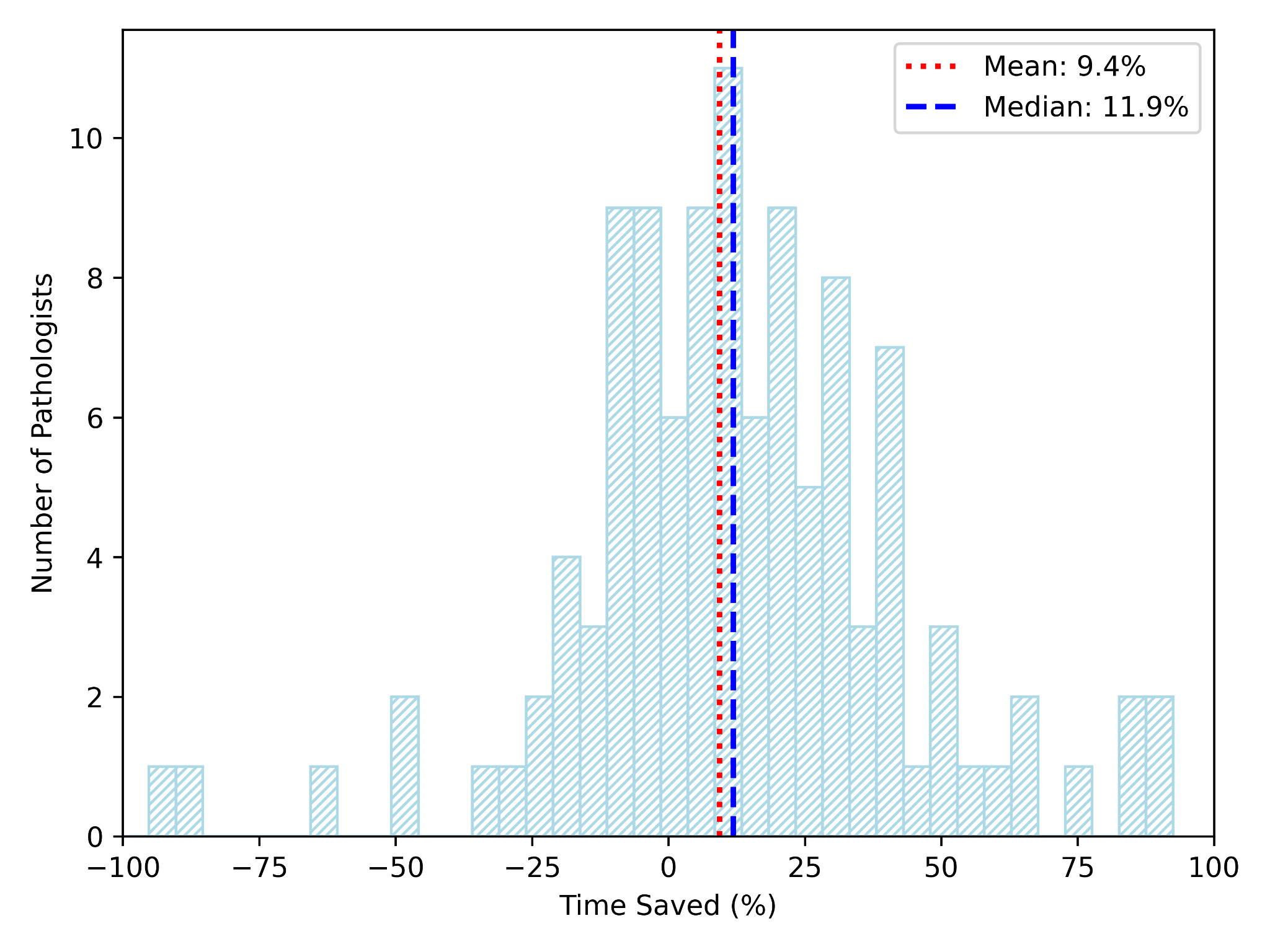


Supplementary Figure 8. Distribution of percentage of time saved with AI aid. This histogram illustrates the distribution of the overall percentage of time saved by pathologists when using the AI aid. Values with positive x-coordinates indicate time savings achieved with the use of AI aid. The figure is zoomed in to show distribution details. A bin width of 5% is used to categorize the time differences. The time savings were determined by the formula: (total time saved/total time spent on conventional assessment) X 100%.
